# White matter structure and myelin-related gene expression alterations with experience in adult rats

**DOI:** 10.1016/j.pneurobio.2020.101770

**Published:** 2020-04

**Authors:** Cassandra Sampaio-Baptista, Astrid Vallès, Alexandre A. Khrapitchev, Guus Akkermans, Anderson M. Winkler, Sean Foxley, Nicola R. Sibson, Mark Roberts, Karla Miller, Mathew E. Diamond, Gerard J.M. Martens, Peter De Weerd, Heidi Johansen-Berg

**Affiliations:** a Wellcome Centre for Integrative Neuroimaging, FMRIB, Nuffield Department of Clinical Neurosciences, University of Oxford, Oxford OX3 9DU, UK; b Department of Molecular Animal Physiology, Donders Institute for Brain, Cognition and Behaviour, Radboud Institute for Molecular Life Sciences (RIMLS), Radboud University Nijmegen, 6525 GA Nijmegen, The Netherlands; c Department of Neurocognition, Faculty of Psychology and Neurosciences, Maastricht University, 6200 MD Maastricht, The Netherlands; d Cancer Research UK and Medical Research Council Oxford Institute for Radiation Oncology, Department of Oncology, University of Oxford, Churchill Hospital, Oxford OX3 7LE, UK; e Department of Cognitive Neuroscience, Radboud University Nijmegen, Donders Institute for Brain, Cognition and Behaviour, 6500 HB Nijmegen, The Netherlands; f Maastricht Centre for Systems Biology (MaCSBio), Maastricht University, 6229 ER Maastricht, The Netherlands; g Tactile Perception and Learning Lab, International School for Advanced Studies (SISSA), 34136 Trieste, Italy

**Keywords:** mRNA expression, White matter, MRI, Myelin, Genome-wide RNA sequencing, Plasticity

## Abstract

White matter (WM) plasticity during adulthood is a recently described phenomenon by which experience can shape brain structure. It has been observed in humans using diffusion tensor imaging (DTI) and myelination has been suggested as a possible mechanism. Here, we set out to identify molecular and cellular changes associated with WM plasticity measured by DTI. We combined DTI, immunohistochemistry and mRNA expression analysis and examined the effects of somatosensory experience in adult rats. First, we observed experience-induced DTI differences in WM and in grey matter structure. C-Fos mRNA expression, a marker of cortical activity, in the barrel cortex correlated with the MRI WM metrics, indicating that molecular correlates of cortical activity relate to macroscale measures of WM structure. Analysis of myelin-related genes revealed higher myelin basic protein (MBP) mRNA expression. Higher MBP protein expression was also found via immunohistochemistry in WM. Finally, unbiased RNA sequencing analysis identified 134 differentially expressed genes encoding proteins involved in functions related to cell proliferation and differentiation, regulation of myelination and neuronal activity modulation. In conclusion, macroscale measures of WM plasticity are supported by both molecular and cellular evidence and confirm that myelination is one of the underlying mechanisms.

## Highlights

• Somatosensory experience results in DTI changes in white matter (WM).
• Macroscale metrics of WM structure correlate with mRNA markers of cortical activity.
• MBP immunohistochemistry and mRNA expression indicate changes in myelin.
• Both molecular and cellular evidence are consistent with DTI findings.
• Myelination is one component of macroscale measures of WM plasticity.

## 1 Introduction

There is accumulating evidence that structural changes in white matter (WM) occur in response to changes in experience, even during adulthood (Sampaio-Baptista and Johansen-Berg, 2017). Neuroimaging studies have reported experience-induced structural WM plasticity in humans (Hofstetter et al., 2013; Scholz et al., 2009). For example, motor skill learning, such as juggling (Scholz et al., 2009) and whole-body balancing tasks (Taubert et al., 2010), have been widely reported to induce changes in diffusion tensor imaging (DTI) metrics. Additionally, somatosensory tasks, like Braille reading training, results in increases in fractional anisotropy (FA) in WM tracts in individuals with normal vision (Debowska et al., 2016). FA is a DTI-derived metric that is modulated by an array of WM features including fibre organization, axon diameter, myelin thickness or length, and changes in astrocyte morphology, among others. Given that MRI measures are nonspecific, cellular and molecular interpretation of the underlying mechanisms is challenging (Sampaio-Baptista and Johansen-Berg, 2017; Zatorre et al., 2012). Recent rodent studies have combined both imaging and immunohistochemistry, to attempt to investigate the underlying cellular mechanisms of macroscale WM plasticity. In response to both cognitive and motor training, higher intensity immunostaining for myelin basic protein (MBP) has been found to colocalize with higher FA (Blumenfeld-Katzir et al., 2011; Sampaio-Baptista et al., 2013), implicating myelination as one of the mechanisms that underlies changes detected by DTI. However, the underlying molecular mechanisms of experience-dependent WM plasticity during adulthood are still unclear, and how they relate to macroscale changes detected by neuroimaging is not fully understood.

Here, we tested whether somatosensory experience in adult rats induces WM plasticity at the macro and the molecular scale by combining neuroimaging and mRNA expression analysis. We trained adult rats to use theirs whiskers to distinguish between surfaces in a texture detection task (TDT) (von Heimendahl et al., 2007). Structural changes in barrel cortex, such as synapse and spine formation (Trachtenberg et al., 2002) have been extensively reported in response to whisker stimulation (Knott et al., 2002), deprivation (Holtmaat et al., 2006) and somatosensory learning (Kuhlman et al., 2014), along with corresponding changes in expression levels of genes, such as BDNF (Rocamora et al., 1996) and synaptophysin (Ishibashi, 2002). More recently, increases in oligodendrocyte numbers and integration in barrel cortex in response to somatosensory enrichment in middle-aged rats have also been detected (Hughes et al., 2018). WM structure was assessed with DTI and immunohistochemistry. Additionally, we analysed mRNA expression of myelin-related genes to support the structural findings and performed unbiased RNA sequencing analysis to further identify putative molecular mechanisms underlying experience-dependent WM plasticity.

## 2 Results

**Fig. 1:**
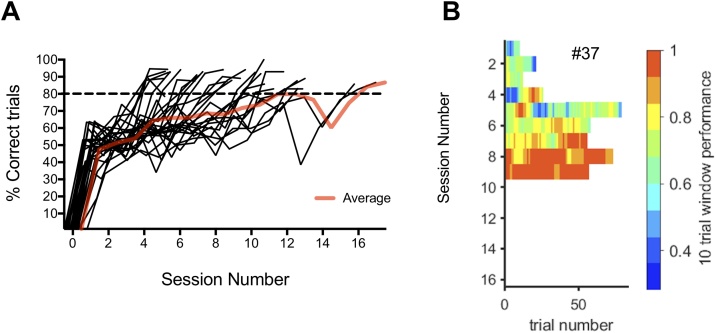
**Texture Detection Task (TDT) Performance. A** Individual performance accuracy (% correct trials) of animals in the TDT group trained on the P100 texture (n = 28). Red line represents average group accuracy. **B** Running average graph of an example rat displaying accuracy per day, with each day represented by a horizontal bar showing color-coded performance levels over 10 trial windows. The total number of trials per session (∼30 min session) tends to increase and performance improves over days. Colour-coding performance scale shown on the right.

Three-month old rats (n = 28) were trained on a texture detection task (TDT) (von Heimendahl et al., 2007) that required a texture identity (rough or smooth) to be associated with a reward side (e.g., turn left on rough, right on smooth). After the initial shaping period, it took the trained animals between 5 and 17 days to reach criterion performance of 2 sessions with > 80 % accuracy (Fig. 1A, B).

## 3 Neuroimaging analysis results

### 3.1 DTI multi-metric analysis revealed WM structural differences between groups and correlations with performance in the texture detection task

To assess effects of experience on WM microstructure we jointly analyzed structural measures calculated from post-mortem DTI scans of left hemispheres from TDT (n = 28) and passive control (PC, n = 20) animals. The PC group was handled daily for a few minutes, without any exposure to the testing setup. We performed a non-parametric combination (NPC) for joint inference analysis, as implemented in PALM, over the 4 DTI measures (fractional anisotropy (FA), mean diffusivity (MD), radial diffusivity (RD) and axial diffusivity (AD)), using the Fisher’s combining function. We tested for a concordant direction of effects across all measures, while allowing the assessment of the significance maps for each measure separately (reported in the partial tests), and as such, inference on which measures would drive a significant joint result, with correction for the multiplicity of tests (Winkler et al., 2016) (see Methods for more details).

**Fig. 2:**
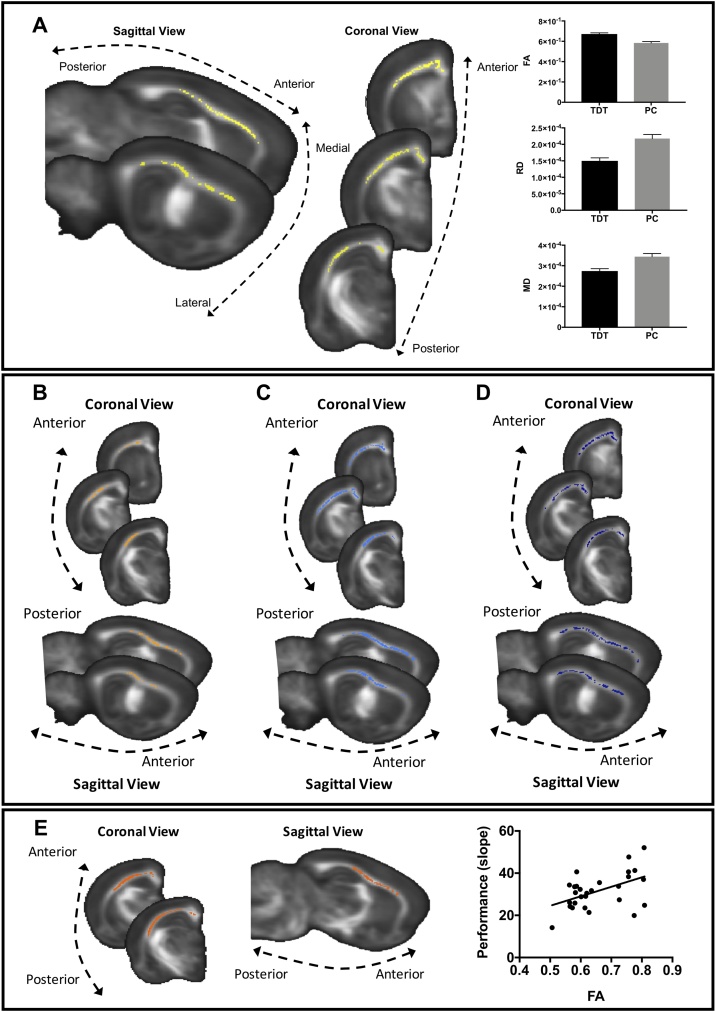
**DTI analysis of WM structure. A** NPC Fisher’s test for joint inference over the 4 DTI measures (FA, MD, RD and AD) (cluster in yellow) was found to be significant p < 0.05 (fully corrected). Bar graphs of FA, RD and MD estimated marginal means (adjusted for the number of exposure days) of the significant yellow cluster are shown to illustrate the direction of differences and not for inference. Error bars represent standard error. **B–D** NPC partial tests. **B** FA (in orange) was significantly higher in the TDT group (p < 0.05, fully corrected). **C** MD (in light blue) was significantly lower in the TDT group (p < 0.05, fully corrected). **D** RD (in dark blue) was significantly lower in the TDT group (p < 0.05, fully corrected). **E** Performance rate correlated with FA (cluster in red) (p < 0.05, fully corrected). Scatter plot showing the correlation between mean FA values of the significant clusters and performance rate is displayed for visualisation of the range of values only and not for inference. In all panels, significant clusters are superimposed on the mean FA template. TDT- Texture detection task group and PC – Passive control.

We first tested for between-group differences across all voxels in the WM skeleton. A significant cluster was found with the NPC joint inference analysis (p < 0.05, fully corrected across all voxels, Fig. 2A) covering a large area of WM under prefrontal and sensorimotor regions (Fig. 2A). These are relevant WM regions for the texture detection task in the light of the barrel cortex’s role in somatosensory information processing (for review see (Feldmeyer et al., 2013)).

Additionally, the NPC partial tests showed that, compared to controls, the TDT group had significantly higher FA (p < 0.05, fully corrected across all voxels and the 4 measures) (Fig. 2B), lower MD (p < 0.05, fully corrected across all voxels and the 4 measures) (Fig. 2C), and lower RD (p < 0.05, fully corrected across all voxels and the 4 measures) (Fig. 2D) across similar areas of WM. AD was not significantly different between groups (p = 0.842, fully corrected across all voxels and the 4 measures).

Secondly, to assess the relationship between task performance and the neuroimaging structural measures, we again used a NPC Fisher’s joint inference analysis to test for voxel-wise correlations between 4 DTI measures and performance rate (slope of the individual curves) across individual animals (n = 28). We did not find a significant correlation between performance rate and the joint 4 DTI measures. However, there was a significant correlation between performance rate and FA (p < 0.05, fully corrected across all voxels and the 4 measures, Fig. 2E) and a trend for RD (p = 0.09, fully corrected across all voxels and the 4 measures, Supplementary Fig. 2) in a similar area of WM (90 % overlap) to that showing group differences (depicted in Fig. 2A). Animals with higher FA (and lower RD) tended to show steeper slopes (i.e. they reached criterion performance with fewer exposure days), suggesting that WM microstructure is related to TDT performance. This correlation could either reflect experience-dependent changes that occurred with task performance, or pre-existing structural differences that relate to performance variation, or a combination of both.

### 3.2 Grey matter MD is lower in the TDT group

**Fig. 3:**
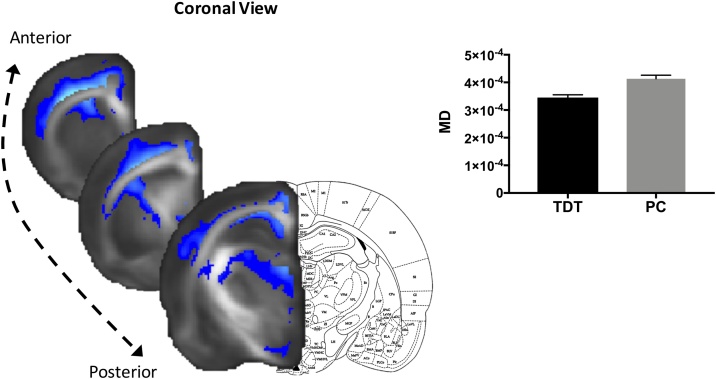
**Grey Matter analysis**. MD (in blue) was significantly lower in the TDT group (p < 0.05, fully corrected). Significant clusters are superimposed on the mean FA template. Bar graph of MD estimated marginal means (adjusted for the number of exposure days) of the significant cluster is shown to illustrate the direction of differences and not for inference. Error bars represent standard error. TDT- Texture detection task group and PC – Passive control.

To assess effects of experience on grey matter (GM) microstructure we tested for MD differences, as this measure reflects water restriction regardless of the structure orientation and can potentially indicate changes in tissue density and/or water content. This analysis revealed clusters with significantly lower MD (p < 0.05, corrected) in the TDT group (n = 28) compared to the PC group (n = 20) (Fig. 3). The significant clusters included both frontal and sensory cortex, hippocampus, and subcortical structures such as striatum and thalamic nuclei.

### 3.3 Learning versus experience

Our main analysis above compared the TDT group to a passive control group in order to identify general experience-dependent effects. To test for specific effects of associative learning versus experience in WM microstructure, we compared a subgroup of TDT rats (TDTsg, n = 12) to an active control group (AC) (n = 12). The AC group was matched for number of training days to a subgroup of TDT rats and were exposed to the same texture discrimination apparatus but were provided with rewards that were not contingent on their response. This allowed the AC group to experience the same textures and similar levels of rewards but without the requirement to differentiate between rough and smooth textures in order to gain the rewards. Rats in TDTsg and AC groups spent the exact same time in the task (for both groups, mean = 8.83 days, S.D. = 3.97).

We used the same statistical approach as above and performed a NPC Fisher’s joint inference analysis as implemented by PALM, over the 4 DTI measures across all voxels in the WM skeleton, using the Fisher’s combining function (Winkler et al., 2016). We tested for differences between the TDTsg (n = 12), AC (n = 12) and PC (n = 20) groups.

**Fig. 4:**
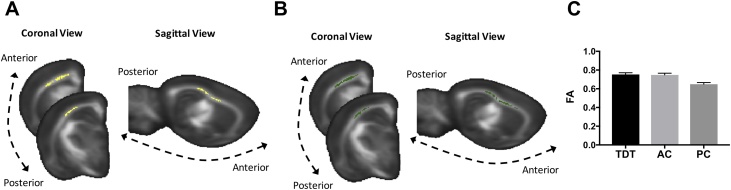
**Effects of learning and experience in WM A** NPC partial tests revealed a trend for higher FA (yellow cluster) in the AC group compared to the PC group (p = 0.1, fully corrected). **B** NPC partial tests revealed a trend for higher FA (green cluster) in the TDTsg group compared to the PC (p = 0.09, fully corrected). No differences or trends were found between the TDTsg group and the AC. **C** Bar graph of FA estimated marginal means (adjusted for the number of exposure days) of the overlapping cluster areas illustrated in A and B. This is shown to illustrate the direction of differences and not for inference. Error bars represent standard error. TDT- Texture detection task group, AC- Active control, PC – Passive control.

The NPC joint inference test did not reveal significant differences between these groups when considering all measures together. However, the partial tests revealed several trends for FA. A trend was seen for higher FA in the AC group compared to the PC group in a cluster underlying barrel cortex (p = 0.1, fully corrected, Fig. 4A, C) and in a very similar cluster for the TDTsg group compared to the PC group (p = 0.09, fully corrected, Fig. 4B, C). There were no significant differences nor trends towards differences between the TDTsg and AC groups (p = 0.71, fully corrected). These results suggest that learning to distinguish between smooth versus rough textures is not necessary for the detected structural differences and that exposure to the task apparatus, whisker stimulation, and rewards, is sufficient to elicit similar structural changes in both active groups compared to a caged group. This indicates that the requirement to differentiate between rough and smooth textures is not necessary to elicit structural WM changes. We cannot exclude that AC rats might have distinguished between contacted textures, even if reward was not contingent on the discrimination.

## 4 Candidate gene analysis and immunohistochemistry results

### 4.1 Somatosensory experience results in higher synaptic c-Fos mRNA expression in the barrel cortex

**Fig. 5:**
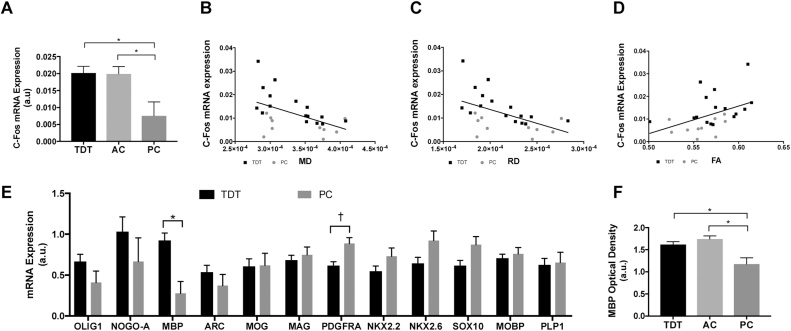
**Candidate genes mRNA analysis and immunohistochemistry**. **A** C-Fos mRNA expression levels of the barrel cortex (estimated marginal means, adjusted for the number of exposure days). **B** Plot of the significant correlation between c-Fos mRNA expression and mean RD of the significant cluster identified in Fig. 2A. **C** Plot of the significant correlation between c-Fos mRNA expression and mean MD of the significant cluster identified in Fig. 2A. **D** Plot of the correlation trend between c-Fos mRNA expression and mean FA of the significant cluster identified in Fig. 2A. **E** MRNA expression levels of myelin-related candidate genes in WM tissue underlying S1. MBP mRNA expression is higher in the TDT group compared to the control group (estimated marginal means, adjusted for the number of exposure days; * p < 0.05, corrected for multiple comparisons;† p = 0.016, uncorrected). **F** Immunohistochemistry revealed significantly higher MBP optical density in the TDT and AC groups (estimated marginal means, adjusted for the number of exposure days). A.u. – Arbitrary Units.

We assessed synaptic c-Fos expression as an indirect marker of cell activity in the barrel cortex to confirm activation of this area in response to the task. As expected, c-Fos mRNA expression was significantly different between groups (One-way ANCOVA; F_(2,33)_ = 12.754; p = 0.000079) (Fig. 5A). Planned comparisons showed a significant difference between TDT and PC (p = 0.000052) and AC and PC (p = 0.000083). No significant differences were found for the comparisons between TDT and AC (p = 0.999). Qualitative analysis of c-Fos in situ hybridization images confirmed the above mRNA expression results (see Supplementary Results, Supplementary Methods, and Supplementary Fig. 3).

### 4.2 Synaptic c-Fos mRNA expression in the barrel cortex correlates with DTI measures of WM microstructure

We further tested for correlations between c-Fos mRNA and the mean FA, MD and RD values of the significant cluster identified in Fig. 2A. We used Bonferroni correction accepting a p-value smaller than 0.0083 as significant.

There were significant negative correlations (Fig. 5B, C, D) across both groups (TDT and PC) between c-Fos mRNA expression and the mean RD (Pearson r = −0.52, p = 0.006, 2-tail), and mean MD (Pearson r = −0.51, p = 0.0078, 2-tail) but not with the mean FA after Bonferroni correction (although a trend for a positive correlation was observed, Pearson r = 0.44, p = 0.024, 2-tail).

Significant negative correlations within the TDT group only were also significant between c-Fos mRNA expression and mean RD (Pearson r = −0.66, p = 0.005, 2-tail), and mean MD (Pearson r = −0.69, p = 0.003, 2-tail) (represented in black in Fig. 5B, C, D).

### 4.3 Myelin basic protein mRNA expression is higher in the WM Co-localised with the barrel cortex of the TDT group

To assess effects of experience on mRNA expression in the WM, the WM underlying the barrel cortex was dissected from a subset of right-brain hemispheres (n = 24; 15 from the TDT group and 9 from the control group) and qPCR was performed on this tissue. Expression of 12 candidate genes known to be centrally involved in myelination was analysed with non-parametric permutation testing as implemented by PALM (Winkler et al., 2016). Of the 12 candidate genes analysed only MBP survived multiple comparisons correction. MBP mRNA expression was found to be significantly higher in the TDT group (p < 0.05, corrected for multiple comparisons, Fig. 5E).

We tested for correlations between mRNA and the mean FA, MD and RD values of the cluster found to be significant in Fig. 2A with Pearson correlation coefficient. No significant correlations were found between the mRNA expression of the candidate list and the DTI measures.

### 4.4 MBP protein expression is higher after somatosensory experience

As MBP mRNA was found to be increased in the TDT group versus the PC group, we processed a subgroup of brains after DTI scanning for MBP immunohistochemistry. Optical density was found to be significantly different between the three groups (TDT, AC and PC) (One-way ANCOVA; F_(2,31)_ = 4.956; p = 0.014) (Fig. 5F). Planned comparisons showed a significant difference between TDT and PC (p = 0.021) and between AC and PC (p = 0.005). No significant differences were found for the comparison between TDT and AC (p = 0.091).

## 5 RNA sequencing results

To gain further insight into the molecular mechanisms underlying the observed structural WM and candidate gene expression differences, we performed an unbiased genome-wide analysis of mRNA expression in WM underlying the barrel cortex, by means of RNA-sequencing in a subgroup of samples (n = 19; including 5 PC, 8 TDT animals and 6 AC).

**Fig. 6:**
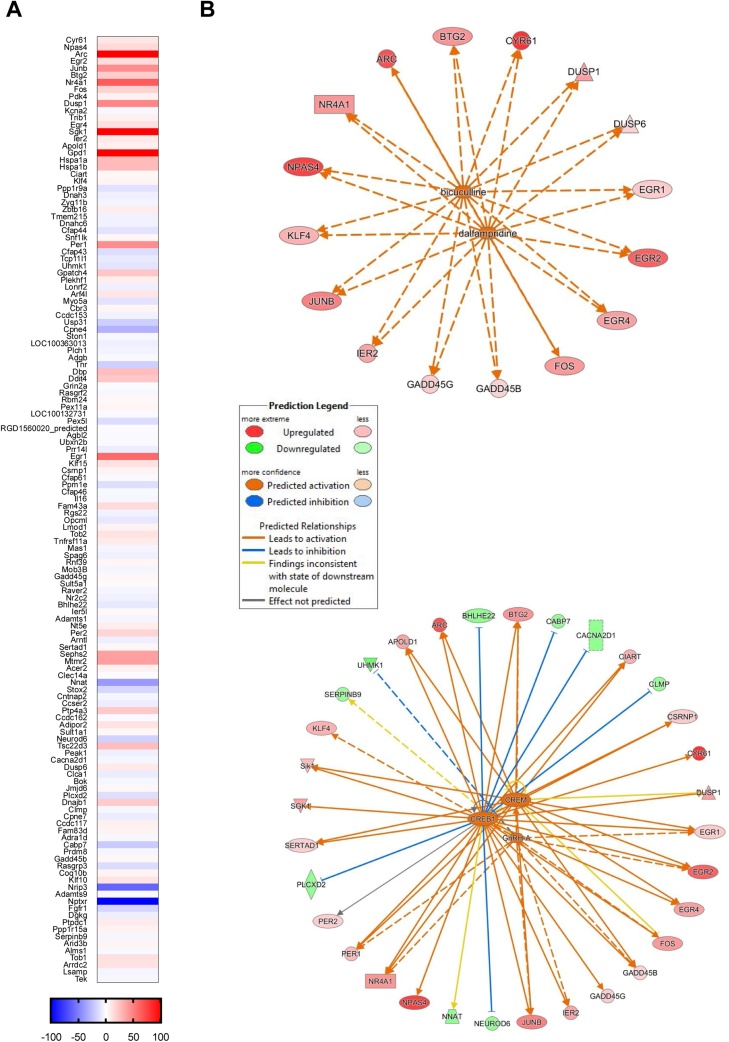
**Genome-wide RNA sequencing results. A** Heat map of 134 significantly differentially-expressed genes identified in the TDT versus PC animals comparison. 124 genes of this list were also significantly differentially expressed between PC and AC. Warm colours indicate significantly upregulated (65 genes) and cold colours indicate significantly downregulated (69 genes). **B** Upstream regulators networks obtained with Ingenuity Pathway Analysis (IPA). Dalfampridine and bicuculline upstream regulator network (top). CREB1, CREM, GnRH-A upstream regulator network (bottom). Relationships between putative upstream regulators and downstream differentially expressed molecules from the RNA sequencing dataset are shown by lines, with solid lines indicating direct relationships, and dashed lines indirect relationships. Colour coding of the lines indicates degree of concordance between predicted and actual direction of regulation. Orange lines indicate predicted activation from the upstream molecules matching observed upregulation of corresponding downstream molecules (red nodes); blue lines indicate predicted inhibition from the upstream molecule matching observed downregulation of the corresponding downstream molecule (green nodes); yellow lines indicate an inconsistent relation of the upstream regulator with the state of the downstream molecule; grey lines represent relationships not predicted by the model. Overall, there is a high concordance (few yellow lines and one grey line) between the predicted and actual direction of regulation of the target molecules by these 5 upstream regulators.

The TDT versus PC comparison led to the identification of 134 differentially expressed genes (likelihood ratio test, p ≤ 5E-6, FC cut-off |1.25|, RPKM cut-off 6) (Fig. 6A, Supplementary Table 1), of which 65 were up and 69 downregulated. From this list of 134 DE genes, 124 genes were also differentially expressed between PC and AC, in the same direction (up or down) and at the same cut-off as in the PC versus TDT comparison. The TDT versus AC comparison lead to the identification of only 6 DE genes (Notch3, Tns1, Zbtb16, Nxn, Yap1, Cfap43), all of them downregulated (Supplementary Table 1).

### 5.1 Gene ontology and ingenuity pathway analysis

In order to interpret the biological significance of the differentially expressed genes, gene ontology (GO) analysis and Ingenuity Pathways Analysis (IPA) were performed. The list of 6 differentially expressed genes (TDT versus AC comparison) led to no findings at the designated thresholds. In contrast, the list of 134 differentially expressed genes (TDT versus PC comparison) yielded several findings that are reported in detail below.

### 5.2 GO analysis: MAPK signalling pathway and transcription regulator activity were enriched

GO analysis identified two significantly enriched terms (corrected p-value ≤ 0.01; Benjamini correction; and at least 5 genes represented in the GO term were considered to be enriched): ‘MAPK signalling pathway’ (with 11 differentially expressed genes) and transcription regulator activity (20 differentially expressed genes) (Supplementary Table 2). MAPK signalling pathway has been implicated in cell proliferation, differentiation and development, and in myelin sheath regulation (Ishii et al., 2012; Zhang and Liu, 2002).

### 5.3 IPA analysis: upstream regulators and networks

To identify molecules upstream of the genes that potentially explain the observed 134 differentially expressed genes, an IPA ‘Upstream Regulators’ analysis was performed (Supplementary Table 3). In simple terms, this analysis uses the IPA database to identify upstream regulators that match the direction of regulation of the downstream differentially expressed molecules from the RNA sequencing dataset (Fig. 6A).

This revealed five predicted upstream regulators showing a high degree of concordance between predicted (by the IPA database) and actual direction of regulation (CREB1, CREM, GnRH-A, dalfampridine and bicuculline) (relationships illustrated in Fig. 6B), all of them with a predicted activated state, an activation z-score > 2.5 and a p-value < 5E-15, with at least 12 target molecules of the differentially expressed list dataset.

CREB1 and CREM had overlapping target molecules (29 target molecules for CREB1, of which 16 were also CREM targets), and had a high degree of overlap with GnRH-A targets (12 targets, of which 9 overlapping with CREB1/CREM targets) (Fig. 6B bottom). CREB and CREM are transcription factors activated by phosphorylation in response to cAMP and other signals (for review see (Mayr and Montminy, 2001)). Both CREB and CREM are involved in regulating the transcription of several genes (c-Fos, BDNF, etc) and have been implicated in neuronal plasticity and memory (Benito and Barco, 2010; Lonze and Ginty, 2002). More recently, CREB has been linked to the transcriptional control of MBP (Meffre et al., 2015), also found to be differentially expressed in our study.

Additionally, two drugs, dalfampridine and bicuculline, shared the same 16 target molecules (Fig. 6B top). Dalfampridine is a broad-spectrum voltage-gated potassium channel blocker that broadens the action potential. It shows beneficial effects in multiple sclerosis patients, probably through restoration of axonal conduction (Dunn and Blight, 2011) and has been shown to promote remyelination after acute nerve injury (Tseng et al., 2016). Bicuculline blocks GABA_A_-mediated inhibition, thereby increasing neuronal activity. This indicates that genes involved in neuronal activity modulation are differentially expressed in our sample, possibly indicating increases or functional changes in axonal activity.

**Fig. 7:**
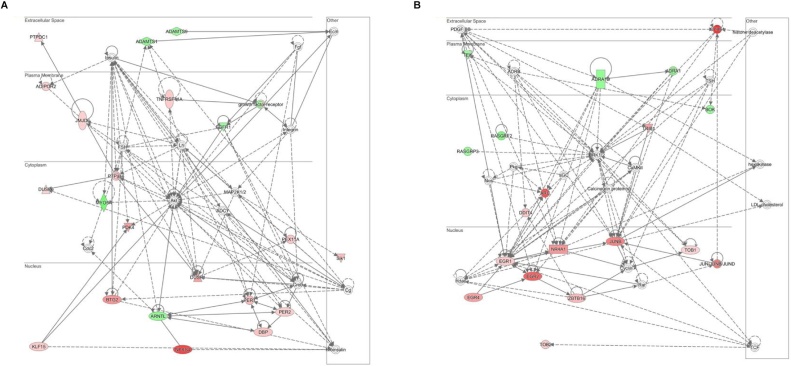
**Two main networks were identified with Ingenuity Pathway Analysis (IPA) *A*** Network 1 with Akt and Creb as hub molecules (where most relationships converge to) **B** ERK1/2 as hub molecules in Network 2. The upregulated differentially expressed molecules from the RNA sequencing analysis are represented in red while the downregulated are labelled in green.

Next we sought to identify functionally related networks of genes and important regulatory hubs (Fig. 7; Supplementary Table 3). There were two networks with a score >30 containing at least 17 molecules of the differentially expressed dataset. In Network 1, two main ‘hub’ molecules (i.e. where most relationships converge to), Akt and Creb (Fig. 7A), were identified, while in Network 2 the main ‘hub’ molecules were Erk1/2 (Fig. 7B). Erk1/Erk2, members of the mitogen-activated protein kinase (MAPK) pathway, and Akt/mTOR, are important regulators of cell survival, proliferation and cell death and are involved in a wide range of disorders including cancer, vascular diseases, Alzheimer’s disease among others (Altomare and Testa, 2005; Mebratu and Tesfaigzi, 2009; Roberts and Der, 2007).

## 6 Discussion

Learning results in macro-level changes in WM that can be detected by DTI measures in both animals (Blumenfeld-Katzir et al., 2011; Sampaio-Baptista et al., 2013) and humans (Hofstetter et al., 2013; Scholz et al., 2009; Taubert et al., 2010). Molecular approaches have identified a number of genomic and proteomic correlates of myelin (de Monasterio-Schrader et al., 2012; Michel et al., 2015; Taylor et al., 2004). The present paper provides insight into how the molecular correlates of myelination relate to macro-changes in WM detected with MRI following behavioural experience. In particular, we have demonstrated that somatosensory experience results in structural white matter plasticity and higher myelination as measured by immunohistochemistry, and we have identified molecular correlates that provide candidate mechanisms underlying these findings, such as myelin formation and/or remodelling.

In addition, our experiments compared the effects of mere exposure to somatosensory stimuli with effects of learning a detection task with those stimuli. Our results suggest that learning the associative task is not necessary for the detected plastic changes and that mere exposure to somatosensory stimulation is sufficient since structural or genome-wide mRNA expression differences between the TDT group and an active control group were not identified.

The WM structural diffusion metrics were found to correlate with barrel cortex synaptic c-Fos expression, suggesting that molecular correlates of cortical activity relate to macroscale measures of WM structural plasticity. Synaptoneurosomes are enriched in synaptic terminals (pre and post) and might also include other cellular (for instance astrocytic) components. Although c-Fos is often regarded as an immediate early gene with exclusive expression in neurons, its expression has also been shown in astrocytes under certain conditions, so we cannot completely exclude non-neuronal contribution to the synaptic c-Fos expression (Herrera and Robertson, 1996).

There is increasing evidence from *in vitro* (Demerens et al., 1996) and *in vivo* (Dutta et al., 2018; Etxeberria et al., 2016; Mensch et al., 2015; Piscopo et al., 2018) studies that neuronal activity modulates myelination, even in adulthood (Gibson et al., 2014). Accordingly, the candidate gene analysis revealed higher MBP mRNA expression, which encodes an essential protein involved in the formation of myelin sheaths, in response to somatosensory experience. This was further supported by immunohistochemistry analysis of MBP. No significant differences were found in the remaining myelin-related genes but PDGFRA mRNA was found to be reduced in the TDT group when using uncorrected statistics. One potential interpretation is that we are mostly detecting formation of myelin at these late stages of learning and task exposure. Current evidence suggests that oligodendrocyte precursor cells (OPCs) differentiation is rapidly triggered by experience. For instance, wheel running triggers OPCs differentiation within just a few hours, peaking at 24 h after wheel exposure (Xiao et al., 2016). A reduction in PDFRA was found after two days of running, indicating OPCs differentiation, and while differentiation is still detected at 8 days post exposure, it is substantially reduced (Xiao et al., 2016). As such, we would expect to detect stronger differences in markers indicating proliferation and differentiation of OPCs at earlier timepoints of task exposure but not at later stages. Given that we wanted to maximize the chances of detecting structural changes with neuroimaging we opted for later timepoints.

The DTI analysis revealed higher FA and lower RD in the TDT group indicating that water diffusion is more hindered across WM tracts after somatosensory experience. Additionally, lower MD was found diffusion in both WM and GM, indicating higher overall restriction of water which could potentially be related to greater tissue density in these areas. While definitive biological interpretation of DTI changes is challenging (Sampaio-Baptista and Johansen-Berg, 2017), this pattern of DTI differences in WM is consistent with cellular mechanisms such as higher myelin thickness or internode length (Etxeberria et al., 2016). Accordingly, we found higher MBP mRNA expression and MBP protein staining intensity suggesting that myelination has been triggered by somatosensory experience. Increases in myelin are consistent with the higher FA and decreased RD found in the current study. These findings are congruent with previous neuroimaging studies that have found higher FA, along with higher MBP immunostaining intensity in response to complex motor and cognitive learning (Blumenfeld-Katzir et al., 2011; Sampaio-Baptista et al., 2013). However, additional mechanisms may also contribute to these findings. For example, changes in axon diameter (Sinclair et al., 2017), nodes of Ranvier length (Arancibia-Carcamo et al., 2017) or axon packing density can potentially also be reflected in these DTI measures. Given the large number of astrocytes present in WM, alterations in this cell population can also potentially modulate DTI measurements (Sampaio-Baptista and Johansen-Berg, 2017). For instance, MD decreases and astrocyte morphological changes have been described in GM in learning paradigms (Blumenfeld-Katzir et al., 2011; Johansen-Berg et al., 2012; Sagi et al., 2012), but there is currently very little understanding of structural contributions of astrocytes to the diffusion signal in the context of long-term experience in WM. Evaluation of the volume, shape and size of astrocytes using immunohistochemistry or other techniques after experience or learning paradigms along with DTI measures would help to clarify in which direction to formulate predictions. The current study used post-mortem DTI of fixed tissue. Tissue fixation can change microstructure properties, and reduces overall diffusivity but anisotropy is preserved (D’Arceuil et al., 2007; Guilfoyle et al., 2003; Sun et al., 2003). Future studies using in vivo MRI would be useful to confirm that these changes are present *in vivo* and to relate microstructural change to physiological measures.

The genome-wide mRNA analysis identified 134 differentially expressed genes that are associated with functions related to neuronal plasticity (CREB1, CREM), memory (CREB1, CREM), neuronal activity modulation (16 target molecules of voltage-gated potassium channels and GABA_A_-mediated inhibition blockers), cell proliferation control, differentiation and protein synthesis (CREB1, CREM, Akt and Erk1/Erk2), and myelin-sheath thickness regulation (Erk1/Erk2). This group of differentially expressed genes indicate that WM has undergone functional and structural plasticity in response to somatosensory experience. In particular, Erk1/Erk2, together with Akt/mTOR, have been proposed as two main signalling pathways for the control of proliferation and differentiation in OPCs and in myelin sheath regulation in adult oligodendrocytes (Bibollet-Bahena and Almazan, 2009; Cui and Almazan, 2007; Dai et al., 2014; Guardiola-Diaz et al., 2012; Snaidero et al., 2014). The Akt and mTOR signalling pathway plays a central role in promoting myelination (reviewed in (Norrmen and Suter, 2013)) and forced activation of the Akt pathway in adult oligodendrocytes results in growth of myelin sheaths (Snaidero et al., 2014). Erk1/Erk2, from the mitogen-activated protein kinase (MAPK) pathway (also significantly enriched in our analysis), is also an important regulator of myelin-sheath thickness in the CNS (Ishii et al., 2012; Jeffries et al., 2016). Conditional upregulation of Erk1/Erk2 results in global increases in myelin thickness by preexisting oligodendrocytes of adult mice, faster nerve conduction velocity and behavioural changes (Jeffries et al., 2016). Furthermore, Erk2 has been described to have an important role in oligodendrocytes in the translational control of MBP (Michel et al., 2015), which is also in line with our findings. As Erk1/Erk2, Akt, MAPK fulfil a variety of general functions in cell survival and protein synthesis, the detected changes could reflect other processes than oligodendrocyte and myelin regulation. Still, the MBP mRNA and MBP immunohistochemistry findings lend further support to the involvement of myelin formation as one component. Overall our findings are compatible with both *de novo* myelin formation by newly formed oligodendrocytes and potential increases in thickness of myelin sheaths by pre-existing oligodendrocytes. Histological assessment of OPCs and mature oligodendrocytes could provide clues on population dynamics, but would not definitively characterize new sheath formation and compaction. Myelin thickness can only be accurately quantified with electron microscopy (EM). Recently, Mitew and colleagues demonstrated with EM that active neurons have thicker myelin. Using immunohistochemistry they also found that active neurons had more internodes created by newly formed oligodendrocytes (Mitew et al., 2018). This study suggests that myelin thickness alterations are associated primarily with new oligodendrocytes but cannot definitively exclude remodeling by pre-existing oligodendrocytes (Mitew et al., 2018). To specifically quantify if myelin thickness alterations in response to experience are associated with newly differentiated oligodendrocytes or with myelin remodeling by preexisting oligodendrocytes is technically challenging and has so far not been assessed in mammals.

In conclusion, somatosensory experience resulted in macroscale structural changes detected with DTI that are consistent with higher myelination. This is supported by MBP immunohistochemistry and molecular evidence of higher MBP mRNA expression, and the expression of genes involved in the regulation of myelin sheath formation and, of proliferation and differentiation of OPCs. Additionally, WM structure correlated with cortical activity as measured by c-Fos mRNA expression, consistent with the idea that cortical experience-dependent mechanisms could trigger WM plasticity. Taken together our results demonstrate that myelination occurs in response to somatosensory experience and that this experience-dependent myelin plasticity is reflected in DTI metrics in WM.

This work paves the way for future studies to examine the specific effects of the identified genes on MRI measures by combining genetic (Jeffries et al., 2016; McKenzie et al., 2014) or pharmacological manipulation in rodents with imaging read-outs. This would allow to precisely identify the molecular and cellular mechanisms which underlie changes in MRI measures of plasticity and could offer important clues to the biological changes underlying imaging signals recorded in humans.

## 7 Material and methods

### 7.1 Animals

All behavioural experiments were conducted at Radboud University Nijmegen (The Netherlands). The experiments were approved by the Animal Ethics Committee of the Radboud University Nijmegen (The Netherlands), according to Dutch legislation and all procedures were performed according to the project and personal licenses held by the experimenters.

60 animals (3 months old, male Long Evans rats (250–450 g) (Harlan, Bicester, UK)) were housed in standard laboratory conditions under a 12-h light/12-h dark cycle at 20 °C temperature and 40–70 % humidity. The animals were housed individually for more precise control of their general welfare and because group housing may interfere with the task experience. All animals were given appropriate time to acclimate after delivery (1 week minimum) and had ad libitum access to food and water. After this period, they were handled daily for one week before the start of the task. Before the task, animals were exposed to the testing arena for 10 min each day for 1–2 days under dim visible light.

The animals were given no access to water for a period of 24 h before the first session. From here on, they received water during the task (0.1 ml of water per correct trial) and water was also made available ad libitum for 30 min after the session. The delay between the end of the task and the time period when water was freely available varied between 30 min to 2 h in order to prevent the animals from learning that water would be available after the testing period.

### 7.2 Texture detection task

The texture detection task (TDT) is based on a previously described task (von Heimendahl et al., 2007). Rats were trained to use their whiskers to distinguish between a smooth and a textured surface using operant conditioning as described below. Training was performed in the dark to avoid the influence of visual cues on performance. Potential olfactory cues were removed from textures by washing them at least once every individual animal session, and by using different sets of identical textures that were interchanged randomly between animals and sessions.

Rats were tested individually. During testing, the animal was placed on a 30 cm elevated platform with two water dispensers on each side. Under this platform was a small bridge where the animal could place its front paws for a short period of time in order to reach the stimulus presented in front of the platform. The stimulus consisted of a series of rectangle shapes with patterns that could be varied depending on the animal’s performance in the task.

During the shaping period the animal was placed on the apparatus, and every appropriate response was rewarded. First, water was randomly delivered in order for the animal to learn where the water was placed. After the animal had learned this, it was rewarded for leaning on the edge of the platform and reaching the stimulus. Finally, water was delivered when the animal touched the texture with its whiskers.

During the training phase of the TDT, the stimulus was either a smooth texture (reference texture) or a positive copy of sandpaper on a resin material. Each animal was trained with a fixed association (e.g., turn left on rough, right on smooth). Only if it approached the correct drinking spout, the animal was given a water reward (0.1 ml per reward); for an incorrect choice, it received no water. The next trial started with a delay of 5 s. Between trials, the texture's stand was turned about its vertical axis by a computer-controlled stepping motor, which allowed for quick, randomized, and automated switching between textures. Each session lasted for about 30 min, during which the animal performed between 60–100 trials.

### 7.3 Experimental design

Animals were randomly assigned to the TDT and control groups, balancing for weight to obtain equal weight averages between the groups.

#### 7.3.1 Texture detection task (TDT) group

For the TDT, animals (total n = 28) were trained to distinguish between the reference texture (smooth) and a P100 texture (162 um average particle diameter). Rats were trained 5 days a week. Individual rats were sacrificed the day after they reached criterion (2 sessions at > 80 % accuracy) on the P100 texture, in order to have comparable performance levels between animals. This resulted in a variation in the number of task exposure days which was then controlled for in subsequent analyses as described below.

A subgroup of rats (n = 8) were further trained to detect increasingly more fine-grained textures after reaching performance criterion in the P100 texture in a stepwise manner: P150 (100 um average particle diameter), P220 (68 um), P280 (52.2 um), P360 (40.5 um), P400 (35 um), P500 (30.2 um) and P600 (25.8 um). When the animals performed above criterion (> 80 % accuracy) for a given texture, the rough texture was changed to a finer one on the following training day. Rats were sacrificed the day after they reached > 80 % accuracy on the P600 texture. After the rats had associated the correct reward side with the first texture, increasing the difficulty of the texture discrimination did not alter their accuracy (Supplementary Fig. 1). Negative control experiments were performed in this subgroup of rats to demonstrate that the animals distinguished smooth vs rough textures and not other sensory attributes of the task (e.g. noises or odours). To do that, animals were presented with the same texture (P400 versus P400) and their performance was assessed. When rats were presented with the same texture their performance accuracy dropped to chance levels (Supplementary Fig. 1).

#### 7.3.2 Active control (AC) group

12 rats were matched to an individual in the TDT group. Rats were water restricted and exposed to the TDT task for the same period of time as the matched animal. However, these animals were rewarded randomly, and not in relation to texture-response contingencies. They received a similar number of rewards as the matched animal throughout the entire training period and were sacrificed after the same number of training sessions as the matched animal in the TDT group.

#### 7.3.3 Passive control (PC) group

Caged controls (n = 20) were handled and weighed daily; their body weight served as a reference body weight with respect to the other group.

### 7.4 Brain preparation

TDT rats were sacrificed by rapid decapitation without anaesthesia on the day after they reached criterion. The AC group were sacrificed after the same number of training sessions as the matched animal in the TDT group. On the day of sacrifice, animals were trained on their respective task for 15 min, then placed back in their home cage for 15 min, after which they were sacrificed and the brains were removed. The PC group was handled for 15 min then placed back in their home cage for 15 min prior to the sacrifice. The right hemisphere was frozen on dry ice and kept at −80 °C for molecular analysis and the left hemisphere was immersed in 4 % PFA for DTI acquisition.

For DTI acquisition, all left brain hemispheres (n = 60) were placed into falcon tubes (50 ml) in pairs (one from each group), one hemisphere above the other, and embedded in 2 % agarose gel (Sigma-Aldrich) (Sampaio-Baptista et al., 2013). The hemispheres were aligned to each other along the posterior – anterior axis.

### 7.5 MRI acquisition

All 60 ex-vivo left brain hemispheres were scanned in pairs overnight with a 4.7 T MRI scanner (Agilent Technology Inc., USA) at Radiobiology Research Institute, Churchill Hospital, Oxford. DTI scanning parameters were as follows: Spin-Echo Multi-Slice Diffusion Weighted (SEMSDW) sequence, b = 2000s/mm^2^, 30 diffusion directions, 4 averages plus 8 images with no diffusion weighting, 40 slices, slice thickness 0.5 mm, field of view 25 × 50 mm, matrix size 96 × 192 (resolution 0.26 × 0.26 × 0.5 mm).

### 7.6 MRI statistical analysis

The data were pre-processed according to standard procedures in FSL (Smith et al., 2004). Tract Based Spatial Statistics (TBSS) (Smith et al., 2006) was applied to the pre-processed data. Images were then analysed as described elsewhere (Sampaio-Baptista et al., 2013). Briefly, all FA maps were aligned with linear and non-linear transformations to the study specific template and averaged to generate the mean FA image, from which the WM skeleton was extracted. The skeleton was thresholded at an FA value of 0.36 to contain only the major tracts (Sampaio-Baptista et al., 2013). Finally, the FA values of the tract centres were projected onto the skeleton for each rat brain and fed into statistical analysis. MD, RD and AD skeleton maps were created with the same method, using the FA registrations and skeleton projections as implemented in TBSS for non-FA images (Smith et al., 2006).

We used Permutation Analysis of Linear Models (PALM) (Winkler et al., 2016) for multi-measures analysis. PALM is a tool that allows inference over multiple modalities, including non-imaging data, using non-parametric permutation methods, similarly to the *randomise* tool in FSL (Winkler et al., 2014), although offering a number of features not available in other analysis software, such as the ability for joint inference over multiple modalities, or multiple contrasts, or both together, while correcting FWER or FDR across modalities and contrasts (Winkler et al., 2016).

We used PALM to assess the joint and individual contribution of the 4 DTI measures while simultaneously correcting across the tests. Non-Parametric Combination (NPC), as implemented in PALM, was used for joint inference over the 4 DTI measures (FA, MD, RD and AD). NPC works by combining test statistics or p-values of separate (even if not independent) analyses into a single, joint statistic, the significance of which is assessed through synchronized permutations for each of the separate tests. The synchronized permutations for the separate tests accommodate, implicitly, any eventual lack of independence among them. Such a joint analysis can be interpreted as a more powerful, permutation-based version of the classical multivariate analysis of covariance (MANCOVA); differently than MANCOVA, however, NPC allows investigation of the direction of joint effects.

Here we used NPC with Fisher’s combining function, testing for effects with concordant directions across the 4 DTI measures. A cluster-forming threshold of t > 1.7 and 5000 permutations were used to determine p-values FWER-corrected for multiple comparisons (across all voxels and the 4 DTI measures). The chosen cluster-forming t threshold was based on the degrees of freedom of the sample. Clusters with a corrected significance of p < 0.05 were deemed significant.

We performed two statistical tests in the WM analysis. First we tested for differences between groups and included the total number of exposure days per animal as a covariate. Second, we tested for correlations between performance rate and the 4 DTI measures. This was calculated by fitting a logarithmic model and extracting the slope of the percentage of correct trials curve for each individual animal (curves are illustrated in Fig. 1A).

Further, we tested for GM differences using MD only as this measure can indicate changes in tissue density regardless of the structure orientation. We tested for group differences and included the total number of exposure days per animal as a covariate. We performed non-parametric permutation testing with the randomise tool as implemented in FSL (Smith et al., 2004), with a cluster-forming threshold of t > 1.7 and 5000 permutations were used to determine corrected p-values. The chosen cluster-forming t threshold was based on the degrees of freedom of the sample. Clusters with a corrected significance of p < 0.05 were deemed significant.

### 7.7 Tissue dissection

A subset of right brain hemispheres was dissected for qPCR and RNA-SEQ. The experimenter was blind to the group for all the following procedures. All procedures were performed under RNase-free conditions. The brain hemispheres were sliced into 300 μm coronal sections using a cryotome (Leica GmbH, Germany) at −15 °C and mounted on glass slides. Cytochrome oxidase-stained reference sections were used as a template to locate the barrel cortex, following stereotactic coordinates (Paxinos and Watson, 1998). Punches of the barrel cortex (n = 37; 16 from the TDT group, 10 from the PC group and 11 from the AC group) and in WM (n = 30; 15 from the TDT group, 6 from the AC, 9 from the PC group) directly underneath it of the right hemisphere were taken using a 1.20-mm micropunch (Harris Inc., UK) and stored at -80 °C before RNA isolation took place.

### 7.8 RNA isolation

The experimenter was blind to the group in all the following procedures and the groups were randomly distributed to ensure equal distribution of groups to avoid any technical bias. Samples were homogenized with a TissueLyser (Retsch GmbH, Germany) in TRIzol® Reagent (Invitrogen Co., USA). RNA was isolated with TRIzol® Reagent (Invitrogen), according to the manufacturers’ protocol. The procedure was modified for small amounts of tissue by using 800 μl of TRIzol® Reagent and adding 1 μl of glycogen (Fermentas Inc., USA). RNA concentration and quality was determined with a NanodropTM ND-1000 spectrophotometer (Thermo Fisher Scientific Inc., USA) and 1 % agarose gel electrophoresis, respectively. The samples were kept at -80 °C until further analysis.

### 7.9 Synaptoneurosome preparation

Synaptoneurosomes were prepared by the method described by (Williams et al., 2009), with some modifications. Brain tissue punches were homogenized with a Teflon-homogenizer (12–14 strokes at 1000 rpm) in 4 mL of homogenization buffer, containing 0.35 M sucrose pH 7.4, 10 mM 4-(2-hydroxyethyl)-1-piperazineethanesulfonic acid (HEPES), 1 mM ethylenediaminetetraacetic acid (EDTA), 0.25 mM dithiothreitol, 8 U/ml RNAse inhibitor and a protease inhibitor cocktail (Roche). Cell debris and nuclei were removed by centrifugation at 1000*g* for 10 min at 4 °C yielding pellet P1 and supernatant S1. The S1 fraction was passed sequentially through a series of filters with decreasing pore sizes of 80, 40 and 10 μm (Millipore). The final filtrate was centrifuged at 2000*g* for 15 min at 4 °C yielding pellet P2 and supernatant S2. Pellet P2 containing synaptoneurosomes was resuspended in 200 μL of homogenization buffer. Enrichment of synaptic components in the synaptoneurosomal fraction was assessed by western blot in control experiments. To assess c-Fos expression in synaptoneurosomes, RNA was isolated using the Trizol method, followed by downstream qPCR.

### 7.10 Quantitative PCR (qPCR)

Prior to cDNA-synthesis, 0.5 μg of each RNA sample was treated with 2 U DNase (Fermentas Inc., USA), in the presence of RiboLockTM RNase Inhibitor (20 U/μl) (Fermentas Inc., USA). For cDNA synthesis, through random priming, the RevertAidTM H Minus First Strand cDNA Synthesis kit (Fermentas Inc., USA) was used, following the manufacturer’s guidelines. Prior to analysis, each cDNA sample was diluted 1/15 with MilliQ water. qPCR reactions were performed with the Rotor-Gene 6000 Series (Corbett Life Science Pty. Ltd., Australia). For each reaction, 2.5 μL of each diluted sample of cDNA was added to a mix containing 6.25 μL 2X MaximaTM SYBR Green qPCR Master Mix (Fermentas Inc., USA), 1 μL of each primer (5 μM) and 1.75 μL MilliQ water. Primers were designed using NCBI Primer-Blast (www.ncbi.nlm.nih.gov/tools/primer-blast/) and synthesized at Sigma-Aldrich (UK). Cycling conditions were 10 min 95 °C followed by 40 cycles of 15 s at 95 °C, 30 s at 60 °C and 30 s at 72 °C. After cycling, a melting protocol was performed, from 72 °C to 95 °C, measuring fluorescence every 1 °C, to control for product specificity.

For the candidate gene analysis of the WM, the following genes were selected for their role in myelin and WM plasticity (PLP1, OLIG1, NOGO-A, MBP, MOG, MAG, PDGFRA, NKX2.2, NKX2.6, SOX10, MOBP, ARC). The c-Fos gene expression in synaptoneurosomes was selected to confirm Barrel Cortex neuronal activation in response to the TDT task. Relative expression of the selected genes of interest in WM and GM was calculated using the two most stably expressed housekeeping genes from a set of three tested candidate genes (ACTB, YWHAZ and CYCA) previously reported to be stably expressed in the brain (Bonefeld et al., 2008) to calculate a normalization factor for each sample. The selected housekeeping genes were found to be highly expressed and stable across samples and treatments. The normalization factor was then used to obtain the relative differences between the samples for each primer pair.

### 7.11 MBP immunohistochemistry

After DTI acquisition, a subset of hemispheres (n = 35; 15 from the TDT group, 9 from the PC group and 11 from the AC group) was selected for immunohistochemistry and the brains were placed in 4 % PFA until being processed for immunostaining. Before sectioning, brain tissue was cryoprotected with 30 % sucrose in PBS to avoid freezing artifacts. The brain hemispheres were sliced into 40 μm coronal sections using a sliding microtome (Microm HM440E; Thermo Fisher Scientific) and preserved in antifreeze solution (30 % ethyleneglycol, 20 % glycerol in sodium phosphate buffer, pH 7.3) at −20 °C until further analysis. Immunohistochemistry was performed with free-floating sections (one every sixth section used). Briefly, sections were washed in PBS, followed by an antigen retrieval treatment in 10 mM citric acid buffer pH 8.5 for 30 min at 80 °C. After two brief washes in PBS, sections were placed into 1 % H2O2 in PBS for 30 min, washed in PBS containing 0.05 % Tween 20 (PBS–T), and incubated in blocking buffer (5 % NGS/NDS/NHS, 1 % BSA, 1 % glycine, 0.1 % lysine, 0.4 % Triton X-100 in PBS) for 1 h. Incubation with the first antibody (anti-MBP, SMI-99, Millipore, diluted 1:500 in blocking buffer) was done overnight at 4 °C. The sections were subsequently washed in PBS–T and incubated with donkey anti-mouse biotinylated antibody (Jackson Laboratories) diluted 1:1000 in blocking buffer for 2–3 h at RT, and washed again in PBS–T. The sections were then transferred to a solution containing avidin–biotin–HRP complex (Vectastain Elite ABC Kit, Vector Laboratories) for 1 h, washed in PBS–T, and stained for 10 min in 0.6 mg/ml diaminobenzidine (Sigma-Aldrich) in PBS containing 0.01 % H2O2 and 0.03 % CoCl2. Special care was taken that all the sections to be compared were stained in parallel for the same amount of time. The sections were then mounted onto microscopic slides in PBS, air dried O/N, dehydrated in graded series of ethanol, cleared in xylene, and coverslipped in Entellan (Sigma-Aldrich). The sections were examined under a Leica DM Fluorescence Microscope and digitized images were obtained with a Leica DFC340 FX CCD camera using Leica IM500 imaging software (Leica Microsystems, Germany). The obtained images were analysed using FIJI version 1.49v, obtaining optical density (OD) measurements in the same ROI (white matter underlying barrel cortex) used for tissue dissection.

### 7.12 Statistical analysis of qPCR mRNA expression

Statistical analysis of WM qPCR data was also performed with PALM (Winkler et al., 2016). We tested for group differences (TDT vs PC) with non-parametric permutation testing with a between groups contrast with total number of exposure days as covariate. A p-value of < 0.05 was deemed significant, corrected for multiple comparisons across the 12 genes of interest.

For the qPCR analysis of the c-Fos gene expression in synaptoneurosomes of the Barrel cortex, a one-way analysis of covariance (ANCOVA) was used to test for differences between groups (TDT, PC, AC), with total number of exposure days as covariate with SPSS. A p-value of < 0.05 was deemed significant and pairwise comparisons were corrected with Sidak.

Statistical analysis of MBP immunohistochemistry was performed with a one-way analysis of covariance (ANCOVA) to test for differences between groups (TDT, PC, AC), with total number of exposure days as covariate with SPSS. A p-value of < 0.05 was deemed significant and pairwise comparisons were corrected with Sidak.

### 7.13 RNA sequencing (RNA-Seq)

A subgroup of samples (n = 13; 5 PC, 8 TDT animals and 6 AC) was used for RNA-Seq, five pools of total RNA (with equal input from each individual sample) were made after Trizol extraction, and were further purified and DNase-treated using Qiagen colums (RNeasy Plus Microkit, Quiagen). The yield of the purified RNA ranged between 1.5 and 2 ug total RNA per pool. The five pools were as follows: (1) passive control (PC) (n = 5 individual samples in pool), (2) TDT Long-exposure (LE) (n = 2 individual samples in pool), (3) TDT Mid-exposure (ME) (n = 3 individual samples in pool), (4) TDT Short-exposure (SE) (n = 3 individual samples in pool) and (5) active control (AC) (n = 6 individual samples in pool). The division of the TDT group into 3 pools of samples was made on basis of the number of days that the animals needed to reach criterion in the behavioural experiment: LE: 9–10 days, ME: 6–7 days, SE: 5 days.

The purified RNA pools were sent for further quality control and RNA-Seq analysis to the Genomic Services Lab of the HudsonAlpha Institute for Biotechnology (AL, USA; http://gsl.hudsonalpha.org/). RNA-Seq with ribosomal RNA (rRNA) reduction was used using standard protocols (depth >45 M pair-end reads per sample), and the resulted raw data were received. Data analyses (alignment and statistical analysis) were performed with GeneSifter (Geospiza, PerkinElmer Inc). We compared the PC group with all three TDT groups pools (LS, LM, LF). For each comparison a p-value (likelihood ratio test) and fold change (FC) was obtained and the following cut-offs were applied: p-value ≤5E-6 in all 3 (PC vs LS, LM, LF) comparisons, FC>|1.25| in at least 2 out of 3 comparisons (with a minimum FC>|1.2|), and expression levels (reads per kilobase million, RPKM) > 6 in at least 1 out of the 4 groups compared. Additionally, to test for specific effects of associative learning versus experience the AC group was also compared to the TDT groups pools and the same cut-offs described above were applied. For validation with qPCR, 19 differentially expressed genes were selected to represent a wide range of expression levels (RPKM ranging from to 8.5–393.2), fold change (from 1.22 to 6.36), and direction of regulation (13 up and 6 downregulated genes). We used independent-sample t-tests (2-tail) to test differences between groups (TDT vs PC). Group differences were found in 16 genes (Supplementary Fig. 4), suggesting that the 134 DE genes carries high validity.

### 7.14 Gene ontology (GO) enrichment analysis and ingenuity pathways analysis (IPA)

Gene Ontology (GO) enrichment analysis of the differentially expressed genes was performed using the web-based gene ontology tool from the Database for Annotation, Visualization and Integrated Discovery (DAVID) 6.7 (http://david.ncifcrf.gov) (Dennis et al., 2003; Huang da et al., 2009a, b). For the enrichment analysis (Functional Annotation Chart tool), default software settings were used, and GO terms with a corrected p-value ≤ 0.01 (Benjamini correction) and at least 5 genes represented in the GO term were considered to be overrepresented (enriched).

Ingenuity Pathways Analysis (IPA) (Ingenuity Systems Inc., USA), was used to perform pathway, network and upstream regulator analyses to explore relationships between genes on the basis of curated information present in the IPA database. For pathway and interaction network analyses, a score was obtained (calculated as the –log of the associated Fisher’s exact test p-value). This score indicates the likelihood that the assembly of a set of focus genes in a network could be explained by random chance alone; networks with scores of 2 or higher have at least a 99 % confidence of not being generated by random chance alone. Upstream regulator analysis generated a list of putative upstream regulators of the differentially expressed genes, and indicate, for each putative upstream regulator, a predicted activation state, activation z-score, p-value of overlap and list of putative target genes of the differentially expressed dataset.

## Declaration of Competing Interest

We report no conflict of interest.
