# Supplementary material for: White matter structure and myelin-related gene expression alterations with experience in adult rats
Source: Prog Neurobiol. 2020 Apr;187:101770. doi: 10.1016/j.pneurobio.2020.101770 (PMC7086231; doi:10.1016/j.pneurobio.2020.101770)
Supplement: Supplementary file 2 [file mmc2.docx]

***Supplementary Fig. 2*** *There is a trend towards a negative correlation between performance rate and RD (cluster in blue) (p = 0.09, fully corrected). Scatter plot showing the correlation between mean RD values of the significant clusters and performance rate is displayed for visualisation of the range of values only and not for inference. Significant clusters are superimposed on the mean FA template.*
