# Supplementary material for: White matter structure and myelin-related gene expression alterations with experience in adult rats
Source: Prog Neurobiol. 2020 Apr;187:101770. doi: 10.1016/j.pneurobio.2020.101770 (PMC7086231; doi:10.1016/j.pneurobio.2020.101770)
Supplement: Supplementary file 3 [file mmc3.docx]

***Supplementary Fig. 3*** *In situ hybridization of the barrel cortex shows high expression of c-fos in Active controls and the texture detection group. Representative slices from A Passive control B Active control C Texture detection group.*
