# Supplementary material for: White matter structure and myelin-related gene expression alterations with experience in adult rats
Source: Prog Neurobiol. 2020 Apr;187:101770. doi: 10.1016/j.pneurobio.2020.101770 (PMC7086231; doi:10.1016/j.pneurobio.2020.101770)
Supplement: Supplementary file 4 [file mmc4.docx]

**Supplementary Fig. 4** **A** *Plot of the 19 genes selected for RNA-seq validation with qPCR. All genes were found to be significantly different between groups except for plch1, Adra1d, and Tnr.* **B** *Table indicating the P-values, degree of freedom (df) and t values for each gene. Error bars represent standard error. TDT- Texture detection task group and PC – Passive control.*
