# Supplementary material for: White matter structure and myelin-related gene expression alterations with experience in adult rats
Source: Prog Neurobiol. 2020 Apr;187:101770. doi: 10.1016/j.pneurobio.2020.101770 (PMC7086231; doi:10.1016/j.pneurobio.2020.101770)
Supplement: Supplementary file 8 [file mmc8.docx]

C-FOS IN SITU HYBRIDIZATION RESULTS

A qualitative analysis of the images revealed very little c-fos mRNA expression in PC animals. A similar expression pattern was observed in both the TDT and AC groups (representative slices shown in Supplementary Fig. 3).

C-FOS IN SITU HYBRIDIZATION METHODS

Before sectioning, brain tissue was cryoprotected with 30% sucrose in PBS to avoid freezing artifacts. The brain hemispheres were sliced into 40 µm coronal sections using a sliding microtome (Microm HM440E; Thermo Fisher Scientific) and preserved in antifreeze solution (30% ethyleneglycol, 20% glycerol in sodium phosphate buffer, pH 7.3) at -20 °C until further analysis. A subset of coronal sections (n=6; 2 from the TDT group, 2 from the PC group and 2 from the AC group) was selected for in situ hybridization. For probe generation, primers were designed using NCBI Primer Blast ([www.ncbi.nlm.nih.gov/tools/primer-blast/](http://www.ncbi.nlm.nih.gov/tools/primer-blast/)) and synthesized (Sigma, UK). DNA templates for the c-fos gene were generated using a nested PCR approach. Antisense digoxigenin (DIG)-labeled probes were generated using an in vitro transcription labeling with T7 polymerase. Labeled probes were checked using a spotblot and compared to a control labeled RNA (Roche). A free-floating method for in situ hybridization with DIG-labeled riboprobes (Valles et al., 2011) was followed. Briefly, sections were washed in PBS, post fixated in 4% PFA and again washed with PBS. Next, sections were permeabilized in 0.2M HCl and washed with PBS. Following a rinse step in DEPC H20, sections were acetylated with 0.1M TEA pH=8.0 with 0.25% acetic acid anhydride. After treating section with 2XSSC (NaC, triNacitrate, MilliQ H2O) sections were transferred to hybridization solution consisting of hybridization mix, 0.5 mg/ml tRNA (Roche Applied Science), DIG-labelled probe (1 ng/μL) and DEPC H2O. After overnight incubation at 58° C sections were washed in 4xSSC and treated with RNaseA at 37°C. Next, sections were rinsed the following concentrations SSC: 2xSSC, 1xSSC, 0,5xSSC at RT and finally 0.1xSSC at 58°C. For immunodetection sections were first washed in TBS buffer (Tris/Hcl, NaCl, MilliQ, pH 7.5) and AP buffer (1M Tris/Hcl, 5M NaCl, 1M MgCl2, pH 9.5) and incubated in anti-DIG-AP (1:5000) in TBS buffer + 0.5% blocking reagent. After rinsing in TBS and AP buffer sections were colored with nitroblue-tetrazolium-chloride/5-bromo-4-chlor-indolyl-phosphate (NBT/BCIP) medium overnight. Coloring was stopped in 0.1 M Tris, 0.01 M EDTA buffer, pH8.0 and sections were mounted on Superfrost Plus slides (Thermo Fisher Scientific) and dehydrated using increasing ethanol concentrations. Finally, sections were fixed briefly with isopropanol and acetone, cleared in xylene and coverslipped with Ethalan.

The sections were examined under a Leica DM 6000B microscope and representative pictures captured with a Leica DFC480 CCD camera using Leica IM500 imaging software (Leica Microsystems).
