# Supplementary material for: White matter structure and myelin-related gene expression alterations with experience in adult rats
Source: Prog Neurobiol. 2020 Apr;187:101770. doi: 10.1016/j.pneurobio.2020.101770 (PMC7086231; doi:10.1016/j.pneurobio.2020.101770)
Supplement: Supplementary file 9 [file mmc9.pdf]

progress in neurobiology

Peer Review Overview

Manuscript Title: White matter structure and myelin-related gene expression alterations with experience in adult rats

Received	29-Jun-2019
1st Decision	27-Aug-2019
1st Revision Submitted	05-Nov-2019
2nd Decision	28-Nov-2019
2nd Revision Submitted	19-Dec-2019
Accepted	24-Jan-2019

1st Decision Letter

Ref: PRONEU_2019_141
Title: White matter structure and myelin-related gene expression alterations with experience in adult rats
Journal: Progress in Neurobiology

Dear Dr. Sampaio-Baptista,

Thank you for submitting your manuscript to Progress in Neurobiology. We have completed the review of your manuscript and a summary is appended below. The reviewers recommend reconsideration of your paper following major revision. We invite you to resubmit your manuscript after addressing all reviewer comments.

When resubmitting your manuscript, please carefully consider all issues mentioned in the reviewers' comments, outline every change made point by point, and provide suitable rebuttals for any comments not addressed.

We look forward to receiving your revised manuscript as soon as possible.

Kind regards,

Aimee Kao, Associate Editor

Sabine Kastner, Editor-in-Chief
Progress in Neurobiology

Comments from the editors and reviewers

Reviewer 1

This reviewer did not consent to publishing their review.

Reviewer 2

Sampaio-Batista et al. studied the mechanisms driving adult WM plasticity by combining DTI and mRNA expression analysis after somatosensory experience. They found that cortical activity in the barrel cortex viewed with c-fos expression correlated with macroscale measures of WM structural plasticity. In turn, analysis of myelin-related genes revealed higher myelin basic protein expression in WM and 134 differentially-expressed genes in the oligodendrocyte lineage and in neuronal activity modulation.The authors conclude that

macroscale WM changes are underlined at least in part by molecular changes in myelin composition.

The study has potential interest though a number of key questions should be addressed with additional experiments.

Major comments :

- The major claim of the manuscript is that experience, not learning, is sufficient to elicit structural changes. However, the results depicted in figure 4 contradict this view. Why? Is it a matter of the sample size (smaller than in figure 2)? This point is key to the credibility of the whole study.
- Changes in gene expression confirm the same idea: experience, not learning, is the major drive of changes. However, these changes poorly correlate with WM myelin changes.
    - The idea in page 18 « *Our results indicate that somatosensory experience may trigger both de novo myelin formation and potentially increases in thickness of myelin sheaths* » is not supported by the data shown in the manuscript. Detailed histological analysis of myelin by immunohistochemistry and electron microscopy are needed to assess this idea.
    - The authors claim that the genome-wide RNA analysis provides evidence of proliferation control, differentiation and protein synthesis in OPCs (CREB1, CREM, Akt and Erk1/Erk2). This interesting possibility should be assessed by evaluating the size of the OPC and oligodendrocyte population in TDT, AC and PC.
- Myelinated axons are also abundant in GM and a thorough gene expression analysis using genome-wide RNA sequencing should include S1 GM.
- Indicate c-fos mRNA expression in AC and significance of the changes as compared to TDT and PC.
  Was c-fos expression increased in WM underlying S1 in TDT and AC?
  Provide histology data showing cells c-fos staining.
- Changes in myelin gene expression are exclusively restricted to an increase in MBP (Figure 5D). How about protein levels ? What is the biological significance of this minor change ?

Minor comments:

- Significance is only indicated in figure 5A. Indicate significance also in figures 2A, 3 and 5A.
- Illustrate graph plotting performance rate versus RD in figure 2E.
- Although not significant it would be appropriate to show the graph of c-fos expression and FA in figure 5.

1st Author Response Letter

Dear Editors,

We are grateful for the thoughtful and constructive comments of the reviewers.

We have substantially revised the manuscript in response to their comments and believe that it has improved in the process.

Where possible, we have included new data (MBP immunohistochemistry and c-fos in situ hybridization) and performed new analysis to respond to the raised points. There are a few suggestions made by reviewers for new data collection that is not possible – for example electron microscopy and GWAS of cortex. We hope that you will recognise that, although potentially interesting, these additional investigations are not necessary to support the conclusions we make here, and as such are outside the scope of the current study

We also respond to each of the points raised in detail below.

Best wishes,

Cassandra Sampaio Baptista and Heidi Johansen-Berg on behalf of all the co-authors

Reviewer 1

This reviewer did not consent to publishing their review, therefore the rebuttal of the authors is not included either.

Reviewer 2

Sampaio-Batista et al. studied the mechanisms driving adult WM plasticity by combining DTI and mRNA expression analysis after somatosensory experience. They found that cortical activity in the barrel cortex viewed with c-fos expression correlated with macroscale measures of WM structural plasticity. In turn, analysis of myelin- related genes revealed higher myelin basic protein expression in WM and 134 differentially-expressed genes in the oligodendrocyte lineage and in neuronal activity modulation.The authors conclude that macroscale WM changes are underlined at least in part by molecular changes in myelin composition.

The study has potential interest though a number of key questions should be addressed with additional experiments.

Major comments:

- The major claim of the manuscript is that experience, not learning, is sufficient to elicit structural changes. However, the results depicted in figure 4 contradict this view. Why? Is it a matter of the sample size (smaller than in figure 2)? This point is key to the credibility of the whole study.
- Changes in gene expression confirm the same idea: experience, not learning, is the major drive of changes. However, these changes poorly correlate with WM myelin changes.

  Response: We don't consider that the Figure 4 results contradict our conclusion that experience alone is sufficient to elicit structural changes. As acknowledged by the reviewer, this figure reports analyses of a subgroup of our training group and as such has less power to detect significant effects but trends echo what we find in the main analysis. Moreover, our analyses across all aspects of the paper consistently pick out a similar pattern of results – with the learning group and active control group not different from each other and having similar effects compared to a passive control.

  For the imaging data: Our main analysis (Fig. 2) shows significant differences between the learning group (TDT) (n=28) and a passive control (PC). We additionally tested for differences between learning and experience with an Active control (AC) group in a smaller sample (n=12). This analysis revealed trends (note these whole skeleton map analysis are extremely conservative, employing non-parametric permutations and fully

corrected statistics) for greater FA for both AC vs PC (p = 0.1) and for TDTsg vs PC (p=0.09) in highly consistent locations for a whole-skeleton analysis, suggesting both learning and experience alone produce similar effects, albeit with lesser statistical significance than an analysis of larger group sizes. By contrast, no significant effects or trends were found anywhere on the skeleton for the TDT vs AC comparison (lowest p = 0.71).

The reviewer is correct that while the comparison between TDT and PC is significant for the full sample it is a trend for the subsample, suggesting that the subsample used here is slightly underpowered.

However, given the similar results between AC vs PC and TDTsg vs PC (the maps are nearly identical), we interpret these findings as suggesting that the AC and the TDT have similar white matter effects compared to the passive control.

As the reviewer points out we find a similar pattern of results in the genome-wide analysis, with a large number of similar genes differentially expressed for TDT vs PC and for AC vs PC, compared to very few genes differentially expressed for TDT vs AC. Again, this suggests to us that AC and TDT have similar effects compared to PC.

We have now also added MBP immunohistochemistry which again shows the same pattern – significantly greater staining in TDT vs PC and in AC vs PC but not in TDT vs AC.

The three different analysis indicate that experience is sufficient to elicit white matter and myelin-related structural and mRNA changes.

- The idea in page 18 « Our results indicate that somatosensory experience may trigger both de novo myelin formation and potentially increases in thickness of myelin sheaths is not supported by the data shown in the manuscript. Detailed histological analysis of myelin by immunohistochemistry and electron microscopy are needed to assess this idea.

Response: We agree that histological analysis would further strengthen the findings.

We have added immunohistochemistry analysis indicating that MBP optical density is increased in the experimental groups:

> "*MBP Immunohistochemistry*
>
> As MBP mRNA was found to be increased in the TDT group versus the PC group, we processed a subgroup of brains after DTI scanning for MBP immunohistochemistry. Optical density was found to be significantly different between groups (F(1,31)=4.956; p = 0.014) (Fig. 5A). Planned comparisons showed a significant difference between TDT and PC (p = 0.021) and between AC and PC (p = 0.0053). No significant differences were found for the comparison between TDT and AC (p= 0.091)."

Unfortunately we will not be able to collect electron microscopy as the tissue has not been prepared for this method. Given that we will not be able to perform EM we have amended the discussion to reflect this:

> "These functions are compatible with both de novo myelin formation and potential increases in thickness of pre-existing myelin sheaths."
>
> "Future studies will be necessary to specifically examine this through a combination genetics tools and electron microscopy (Mitew et al., 2018)."

- The authors claim that the genome-wide RNA analysis provides evidence of proliferation control, differentiation and protein synthesis in OPCs (CREB1, CREM, Akt and Erk1/Erk2). This interesting possibility should be assessed by evaluating the size of the OPC and oligodendrocyte population in TDT, AC and PC.

  Response: Proliferation and differentiation of oligodendrocytes is one among several possible interpretations of our genome-wide results. We consider that definitive interpretation of the gene expression findings is outside the scope of the current study but could be explored in future studies with that specific aim.

  We have added the following to the discussion:

  > “These functions are compatible with both de novo myelin formation and potential increases in thickness of pre-existing myelin sheaths. However, as Erk1/Erk2, Akt, MAPK fulfil a variety of general functions in cell survival and protein synthesis, the detected changes could reflect other processes than oligodendrocyte and myelin regulation. Still, the MBP mRNA and MBP immunohistochemistry findings lend further support to the involvement of myelin change as one component. Future studies will be necessary to specifically examine this through a combination genetics tools and electron microscopy (Mitew et al., 2018).”

  Myelinated axons are also abundant in GM and a thorough gene expression analysis using genome-wide RNA sequencing should include S1 GM.

  Our main hypotheses for these experiments were related to white matter. Due to the cost of the technique we focused our genome-wide analysis exclusively on white matter.

- Indicate c-fos mRNA expression in AC and significance of the changes as compared to TDT and PC.

  Response: We have now added these results. Consistent with other measures, we found significantly greater c-fos mRNA expression in barrel cortex for TDT vs PC and AC vs PC but not for TDT vs AC.

  > “We assessed synaptic C-FOS expression as an indirect marker of cell activity in the barrel cortex to confirm activation of this area in response to the task. As expected, c-FOS mRNA expression was significantly different between groups (One-way Ancova; $F(2,33)=12.754$; $p = 0.000079$) (Fig. 5A). Planned comparisons showed a significant difference between TDT and PC ($p = 0.000052$) and AC and PC ($p = 0.000083$). No significant differences were found for the comparisons between TDT and AC ($p = 0.999$).”

- Was c-fos expression increased in WM underlying S1 in TDT and AC? Provide histology data showing cells c-fos staining.

  Response: We did not perform c-fos mRNA analysis of WM. We have added results of c-fos in situ hybridization of the barrel cortex in a supplementary figure (Supplementary Fig. 3) and supplementary methods. Qualitative consideration of these figures demonstrate that c-fos expression in barrel cortex is higher in both the TDT and AC groups compared to the PC group, consistent with the mRNA c-fos analysis.

- Changes in myelin gene expression are exclusively restricted to an increase in MBP (Figure 5D). How about protein levels ? What is the biological significance of this minor change ?

Response: We have now added histological analysis of MBP and found significant group differences. These results have been added to the manuscript (respective methods have been added to methods section):

> "*MBP Immunohistochemistry*
>
> As MBP mRNA was found to be increased in the TDT group versus the PC group, we processed a subgroup of brains after DTI scanning for MBP immunohistochemistry. Optical density was found to be significantly different between groups (F(1,31)=4.956; p = 0.014) (Fig. 5A). Planned comparisons showed a significant difference between TDT and PC (p = 0.021) and between AC and PC (p = 0.0053). No significant differences were found for the comparison between TDT and AC (p= 0.091)."

Minor comments:

- Significance is only indicated in figure 5A. Indicate significance also in figures 2A, 3 and 5A.

  Response: We now have added asterisks for figure 5A.

  The statistics are performed in the MR maps with a voxel-wise approach, as such the significant imaging results are represented by the MR maps themselves. The plots are only a representation of the mean values across all voxels within the significant clusters. These are for visualisation of the direction of the group differences and no statistics are performed on these extracted mean values as this would represent 'double-dipping'. As such we do not put asterisks in the graphs to avoid confusion and we explain these are not for inference:

  > "Bar graphs of FA, RD and MD estimated marginal means (adjusted for the number of exposure days used as a covariate in the model) of the significant yellow cluster are shown to illustrate the direction of differences and not for inference. Error bars represent standard error."

- Illustrate graph plotting performance rate versus RD in figure 2E.

  Response: We have added a supplementary figure representing the described trend and plotted the corresponding correlation represented by the mean RD values and performance scores.

  "Supplementary Fig. 2 There is a trend towards a negative correlation between performance rate and RD (cluster in blue) (p = 0.09, fully corrected). Scatter plot showing the correlation between mean RD values of the significant clusters and performance rate is displayed for visualisation of the range of values only and not for inference. Significant clusters are superimposed on the mean FA template."

- Although not significant it would be appropriate to show the graph of c-fos expression and FA in figure 5.

  Response: We have added this graph to Figure 5 and it's now 5D.

2nd Decision Letter

Ref.: Ms. No. PRONEU_2019_141R1
White matter structure and myelin-related gene expression alterations with experience in

adult rats
Progress in Neurobiology

Dear Dr. Sampaio-Baptista,

Thank you for submitting your manuscript to Progress in Neurobiology. We have received comments from reviewers on your manuscript. Your paper should become acceptable for publication pending suitable minor revision and modification of the article in light of the appended reviewer comments.

When resubmitting your manuscript, please carefully consider all issues mentioned in the reviewers' comments, outline every change made point by point, and provide suitable rebuttals for any comments not addressed.

To submit your revised manuscript go to https://www.editorialmanager.com/proneu/ and log in as an Author where you will see a menu item called 'Submission Needing Revision'.

Please resubmit your manuscript by Jan 27, 2020.

We look forward to receiving your revised manuscript.

Yours sincerely,

Aimee Kao
Associate Editor

Sabine Kastner
Editor-in-Chief
Progress in Neurobiology

Comments from the Editors and Reviewers:

Reviewer 1

This reviewer did not consent to publishing their review.

Reviewer 2

The revised manuscript includes now some new data and explanations. However, the authors failed to address major criticisms in my previous comments by doing additional experiments including a detailed histological analysis of the myelin and oligodendrocyte lineage changes. Instead, they provide interpretations of their initial results. I think this is not sufficient.

In my opinion, this paper in its current form does not meet the standards of Progress in Neurobiology.

2nd Author Response Letter

Reviewer 2

We are pleased to see that Reviewer 2 recognised that we provided new data and explanations. Reviewer 2's only remaining issue is: "the authors failed to address major

criticisms in my previous comments by doing additional experiments including a detailed histological analysis of the myelin and oligodendrocyte lineage changes."

In R2's original review a large number of additional experiments and analyses were requested. We were able to perform many of these – such as addition of MBP immunohistochemistry and c-fos in situ hybridisation – but some additional experiments were not possible.

Specifically, in the original reviews, R2 stated that:

"This interesting possibility should be assessed by evaluating the size of the OPC and oligodendrocyte population in TDT, AC and PC."

"Detailed histological analysis of myelin by immunohistochemistry and electron microscopy are needed to assess this idea."

We assume that these are the points that R2 is referring to here.

First, and most importantly - we would argue that the conclusions we are making do not depend on the results of these additional experiments. It is always possible to suggest additional experiments – the key question is whether the conclusions of the current paper require those experiments – and in our view in this case they do not. Our primary focus was on relating novel mRNA measurements to MRI metrics in white matter. All of the data that we have (MRI, mRNA, MBP immunohistochemistry, c-fos in situ hybridisation) supports the claims that we are making – that experience-dependent changes detected in white matter with macroscale non-invasive techniques are associated with changes in myelin markers. While future studies might aim to track down the precise mechanisms that underlie the myelin changes that was not our intention.

In addition – to undertake the suggested experiments would require substantial time and resource as the entire experiment would need to be repeated. We did not give EDU (or some other proliferation marker) to the animals prior to the experiments, which would be fundamental to assess proliferation/differentiation of the OPC and OLs population. Therefore, performing these analyses would require us to re-run the entire experiment. Although, histological assessment of OPCs and mature oligodendrocytes could provide clues on population dynamics, they would not definitively characterize sheath formation and compaction.

Additionally, as explained in our response, the tissue was not processed for EM. Therefore, to satisfy the reviewer's request would require for the experiments to be repeated and tissue to be processed for EM and then quantitatively analysed. EM would allow us to measure myelin thickness but would not allow us to distinguish between new myelin sheaths by recently differentiated oligodendrocytes and "remodelling" of myelin sheaths by pre-existing oligodendrocytes. This is a technical challenge and we are currently not aware of any studies that have been able to perform this experiment in mammals in the context of experience-dependent plasticity.

In summary, the requested additional experiments are not essential for the conclusions we wish to make and are also simply impossible due to the substantial time and resource this would require. However, while we strongly argue that these investigations are outside the scope of our paper – we do recognise that they would provide interesting insights. We are therefore happy to further highlight these questions as potential future research directions and have added the following to the discussion:

“Overall our findings are compatible with both de novo myelin formation by newly formed oligodendrocytes and potential increases in thickness of myelin sheaths by pre-existing oligodendrocytes. Histological assessment of OPCs and mature oligodendrocytes could provide clues on population dynamics, but would not definitively characterize new sheath formation and compaction. Myelin thickness can only be accurately quantified with electron microscopy (EM). Recently, Mitew and colleagues demonstrated with EM that active neurons have thicker myelin. Using immunohistochemistry they also found that active neurons had more internodes created by newly formed oligodendrocytes (Mitew *et al.*, 2018). This study suggests that myelin thickness alterations are associated primarily with new oligodendrocytes but cannot definitively exclude remodeling by pre-existing oligodendrocytes (Mitew *et al.*, 2018). To specifically quantify if myelin thickness alterations in response to experience are associated with newly differentiated oligodendrocytes or with myelin remodeling by preexisting oligodendrocytes is technically challenging and has so far not been assessed in mammals.”
